# An Alien in the Group: Eusocial Male Bees Sharing Nonspecific Reproductive Aggregations

**DOI:** 10.1093/jisesa/iev107

**Published:** 2015-10-30

**Authors:** C. F. dos Santos, M. J. Ferreira-Caliman, F. S. Nascimento

**Affiliations:** ^1^Departamento de Biodiversidade e Ecologia, Faculdade de Biociências, Pontifícia Universidade Católica do Rio Grande do Sul, Avenida Ipiranga, 6681, Porto Alegre, Rio Grande do Sul, Brazil; ^3^Departamento de Biologia, Faculdade de Filosofia, Ciências e Letras de Ribeirão Preto, Universidade de São Paulo, Avenida Bandeirantes, 3900; Ribeirão Preto, São Paulo, Brazil

**Keywords:** Apidae, Behavior, Chemical Ecology, Hymenoptera, Phylogeny

## Abstract

Sexual selection predicts that individuals competing for access to sexual partners should maximize their chances of mating by looking for sites where the chances of finding partners are more likely to occur. However, males of stingless bees have been observed sharing nonspecific reproductive aggregations. This uncommon behavior appears to confer no obvious increase of individual fitness. It has been suggested that this reproductive strategy is due to the similarity between male odors common to different stingless bee species. Cuticular hydrocarbons (CHCs) are candidate odors of interest because their nonvolatile pheromone nature allows them to play an important role in sexual behavior and species recognition. Here, we review the literature to evaluate whether any phylogenetic patterns exist among male stingless bees that aggregate with closely or distantly related species. We also compared the CHC profiles of males of Neotropical stingless bee species (*Plebeia* sp. Schwarz, *Trigona spinipes* (F.), *Tetragona clavipes* (F.), *Nannotrigona testaceicornis* (Lepeletier), *Scaptotrigona* aff. *depilis* (Moure), *Tetragonisca angustula* (Latreille), and *Melipona subnitida* (Ducke) to reveal any chemical similarities among their male odors. We found males of 21 stingless bee species involved in interspecific interactions mainly from Neotropical and Indo-Malayan/Australasian regions. Alien males did not necessarily visit host aggregations of closely related species. Furthermore, the CHC profiles of different studied species were very distinct from each other and do not overlapped at all. It is unclear yet why this apparently nonadaptive behavior carried out by some stingless bee males.

Many male insects aggregate near nests or other sites where sexually receptive virgin females can be found, and where mating usually occurs (Emlen and Oring 1977, Sullivan 1981, Thornhill and Alcock 1983, Sivinski and Petersson 1997). One would expect that such reproductive aggregations would only contain individuals from the same species. In this way, sexual selection could maximize the individual fitness due to the abundance of members of the opposite sex of their species (Darwin 1859). However, while selective pressure on organisms should always optimize the search for sexual partners on species-specific aggregations to avoid any possibility of interspecific mating, this is not always the case. For example, although rare, mixed mating swarms in insects have been well documented in ants, bees, and wasps (Leprince and Francoeur 1986, Nadel 1987, O’Neill 1994, Koeniger and Koeniger 2000, Starr and Vélez 2009, Bänziger and Khamyotchai 2014, Santos et al. 2014).

Particularly in bees, this issue has been rather well illustrated. For example, it has been observed in mixed aggregations of males of three different *Centris* species (Centridini oil bees) predominantly compounded by *Centris decolorata* males, and also by males and females of *C. smithii* and *C. lanipes* (Starr and Vélez 2009). In honeybees (Apini) and stingless bees (Meliponini), some males (or drones) may visit aggregations of other bee species, as well. For example, *Apis cerana* drones shared *Apis dorsata* drone congregation area and vice versa (Koeniger and Koeniger 2000). Yet, in stingless bees, alien males of several species have been observed gather in nonspecific (host) reproductive aggregations (Schwarz 1948; Nogueira-Neto 1954; Kerr et al. 1962; Cortopassi-Laurino 1979, 2009; Bänziger and Khamyotchai 2014; Santos et al. 2014). These examples in bees show us an uncommon sexual behavior, since it is expected that males of only the same species would aggregate for reproductive purposes.

To understand a little more about possible causes for these uncommon sexual behaviors assumed by bee males, we discuss it here using stingless bees as model due rather common occurrence in this bee group. The mating system in stingless bees is lek-like because males congregate on a communal display area for the sole purpose of mating (Emlen and Oring 1977). However, the mating system can diverge from Emlen and Oring’s definition because sexually receptive females (virgin queens) of stingless bees do not directly choose a certain male according to his status position or resource defense within aggregation. Instead, males must be able to pursue and achieve virgin queens during nuptial flight before their competitors (Roubik 1990).

The male aggregation in stingless bees appears to be initiated by the presence of virgin queens within the nest, which can promote the arrival of dozens, or even hundreds of conspecific males. This progressively forms a large aggregation of individuals that wait close to the nest for the nuptial flight of virgin queens (Michener 1946, Kerr et al. 1962, Roubik 1990, Velthuis et al. 2005, Cortopassi-Laurino 2007). At the moment of copulation, male stingless bees are involved in a suicidal mating because their seminal vesicles and genitalia remain attached within the female reproductive tract (Kerr et al. 1962). It not only kills them but also effectively prevents subsequent copulations by other males. Such behavior then becomes obligatory monogamist males and stingless bee queens essentially monandrous, according to observational and molecular data (Kerr et al. 1962, Strassmann 2001, Jaffé et al. 2014).

Selective pressure over stingless male bees can be still stronger. These individuals are involved in a competition among themselves to present better physical vigor, visual perception and chemical discrimination to detect virgin queens during their nuptial flights (Roubik 1990). It has been believed, then, to result in a strong selective pressure on these physiological, anatomical, and behavioral traits (Roubik 1990). Therefore, visiting and staying in a reproductive aggregation compounded of other stingless bee species, as eventually can occur does not seem to be a good competitive strategy.

Some hypotheses could be raised to explain this uncommon male behavior in social bees. First, males of multiple bee species could be attracted by a common highly volatile chemical compound released by the queens. In honeybee species, studies demonstrated that nonsympatric and unspecific drones of *A. cerana*, *A. dorsata,* and *A. florea* can be attracted by the main sex queen pheromone of honeybees (*A. mellifera*), (E)-9-oxodec-2-enoic acid (Butler et al. 1967, Sannasi and Rajulu 1971, reviewed by Koeniger and Koeniger 2000). Second, males of multiple bee species could be attracted to a same local due to the odor released inside colonies. It was proposed by Velthuis et al. (2005) to explain why males of multiple stingless bee species often are seen aggregating themselves together. However, it is not clear which individuals could be potential releasers of such chemical compounds inside colonies. Third, males of different bee species share chemical profiles based on nonvolatile cuticular compounds. This hypothesis was suggested by Kerr et al. (1962) to explain why stingless bee males (alien species) could mistakenly join others males (host species) during reproductive aggregations.

Since male stingless bees may interact with each other in aggregations such as performing antennation or trophallaxis (Cortopassi-Laurino 2007, Santos et al. 2014, Supp Video 1 [online only]), it creates a scenario where they could recognize other species through profiles of cuticular hydrocarbons (CHCs). The CHCs are chemical compounds that have evolved primarily to provide a water-impermeable layer over an insect’s epicuticle to protect them from desiccation and to act as a barrier against microorganisms (Wigglesworth 1957, Hadley 1994). Furthermore, these compounds have assumed multiple functions as signals of colony membership, age, sex, caste, and species, mainly in social species (Blomquist et al. 1998; Dani 2006; Nunes et al. 2008; Provost et al. 2008; Tannure-Nascimento et al. 2008; Ferreira-Caliman et al. 2010, 2013; Nascimento and Nascimento 2012). Therefore, CHCs can be considered ideal candidates to find any chemical similarity among males of multiple stingless bee species.

To date, no studies have been conducted to compare the male odors of different stingless bee species and to determine whether male CHC profiles are sufficiently similar among different species to explain why some males join nonspecific aggregations. That approach may offer opportunities to further our understanding of the evolution and maintenance of this lek-like mating system and offer parallels with the male aggregation behaviors observed in other insect orders (Sullivan 1981, Thornhill and Alcock 1983, Sivinski and Petersson 1997).

Here, we review the literature to find any phylogenetic pattern in the occurrence of male stingless bees visiting nonspecific aggregations. Second, we compared the CHC profiles of stingless bee males of different species. Our main goals were to organize this knowledge, i.e., whether males of different stingless bee species have been aggregated together and whether CHC profiles could give us any indication of a likely chemical similarity among species.

## Materials and Methods

### 

#### Interspecific Male Interactions

We reviewed the literature dealing with alien males participating in other host male reproductive aggregations of stingless bee species. To visualize these data, we used the Cytoscape software (Shannon et al. 2003). In Cytoscape, we used the Network Analyzer function. After that, we generated styles of networks to link the number of direct edges (i.e., whether there was an interaction between species’ pairs and its direction). Then, data visualization was done for mapping node size ascribing low values to small sizes (few interactions) and high values to greater interactions between alien and host species. Subsequently, we applied workflow function in Cytoscape followed by the group attributes layout and, finally, wordcloud plug-in where all words (here, species’ names) were calculated proportionally according to their frequency in the input text (Saito et al. 2012). Thus, this approach allowed us to integrate and to connect possible relationships according to the data reported in the literature.

During visual data making and by considering its respective interaction (from alien to host species) according to literature, we organized the stingless bee species according to their distribution from Neotropical, Afrotropical, and Indo-Malayan/Australasian regions.

#### Chemical Profiles

Ten males of every *Plebeia* sp. Schwarz, *Trigona spinipes* (F.), *Tetragona clavipes* (F.), *Nannotrigona testaceicornis* (Lepeletier), *Scaptotrigona* aff. *depilis* (Moure), *Tetragonisca angustula* (Latreille), and *Melipona subnitida* Ducke stingless bee species were stored separately in glass vials and kept at 4°C prior to the chemical analysis. All of the species were collected during reproductive aggregations between August 2009 and April 2012, in São Paulo state, Brazil, except for *M. subnitida* that was collected in Rio Grande do Norte state, Brazil. Voucher specimens were deposited at Coleção Entomológica Paulo Nogueira-Neto from Bee Lab Universidade de São Paulo, in São Paulo/Brazil.

The cuticular compounds of each male bee were extracted in 1 ml hexane for 1 min. After evaporation of the solvent under a stream of N_2_, the nonpolar extract was resuspended in 100 µl of hexane and 1 µl sample was injected into a combined gas chromatography-mass spectrometry system (CGMS, Shimadzu, model QP2010, Kyoto, Japan). The separation was carried out on a 30 m Rtx-5MS column with helium as the gas carrier at a flow rate of 1.0 ml min^−^^1^. The oven temperature was initially set to 150°C, and then increased by 3°C min^−^^1^ until it reached 280°C (hold time = 15 min). Analyses were performed in splitless mode. The mass spectra were obtained by 70 eV ionization.

Mass spectra were analyzed using GCMS Solutions for Windows version 2.6 (Shimadzu Corporation, Brazil). The chemical compounds were identified based on their mass spectra by comparison with Nist Library 08 data and with a standard solution of different synthetic hydrocarbons (Fluka).

#### Data Analysis: Male Aggregation Analysis

First, we did a binomial test to evaluate whether stingless bee males aggregate themselves with their own species or not. If so, we estimated the probability on whether there could be a significantly higher number of cases of males from closely related species (here, same genus) participating in a nonspecific aggregation than by chance. For both cases, we applied a binomial test by using the function “binom.test” from stats package for *R* (R Core Team 2014). In the first case, the success number was considered as the number of observations of aggregations receiving alien species versus the total amount of male aggregations observed in literature. In the second case, the success number was considered as the number of observations of males visiting aggregations of closely related species versus distantly related species. For both, we hypothesized a probability of success of 0.8, an alternative hypothesis as two-sided and a confidence interval of 0.95 (95% CI). Data were visualized using Euler diagram provided by venneuler package (Wilkinson 2011) for *R*.

#### Data Analysis: Chemical Analyses

The relative abundance of each compound was estimated from the proportion of the peak area of the total ion chromatograms. We compared the chemical composition of the cuticular surface lipids of males using nonparametric multivariate analyses using PAST version 3.04 (Paleontological Statistics Software Package, Hammer et al. 2001). We performed a nonmetric multidimensional scaling (NMDS) analysis based on the Bray-Curtis distances to generate a graphic showing the differences between species. Bray-Curtis distances were also used to plot a cluster analysis and to make a one-way analysis of similarities (ANOSIM) with 9,999 permutations to test for significance in dissimilarities between the chemical profiles of males of different species.

*R*-value is based on similarity matrices (or distances) ordering the values to perform the statistical procedure. *R*-values between 0 and 1 indicated the level of similarity, where *R* = 0 indicates no difference between the groups and *R* = 1 indicates a larger similarity within a group than between groups. *P* values were adjusted using the Bonferroni correction. Similarity percentages (SIMPER) were calculated to identify the compounds that predominantly contributed to the Bray-Curtis dissimilarities among species.

## Results

### 

#### Alien Males in Host Aggregations

**Fig. 1.**
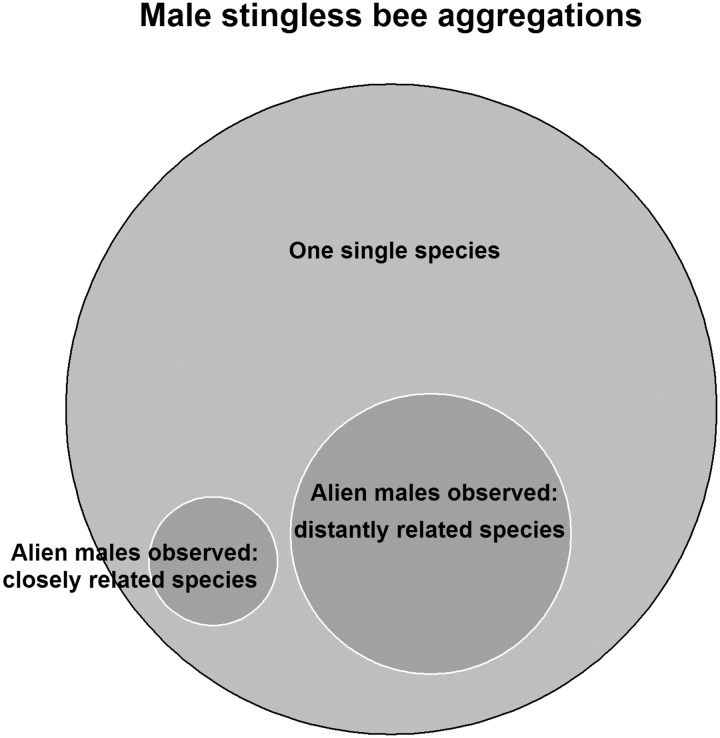
Euler diagram showing male stingless bee aggregations compounded by one single species and relative proportion of those aggregations where males of other stingless bee species have been seen visiting closely or distantly related species.

Our review found at least 65 reproductive aggregations of stingless bees (Supp Table 1 [online only]) of which ∼71% received only one single species, and 29% received males of different stingless bee species at any time (Fig. 1). There was a significant difference between those aggregations formed by only one species and with those having two or more species (Exact Binomial Test, CI: 0.18–0.41; *P* < 0.0001). In these later kind of aggregations (two or more species), just a few percentage of alien males of stingless bee species (circa 18%, Fig. 1) were phylogenetically closely related to the host species. Finally, our binomial test showed that the probability of males joining aggregations of another stingless bee species is 21%, and may occur by chance (Exact Binomial Test, CI: 0.06–0.45: *P* < 0.0001).

**Fig. 2.**
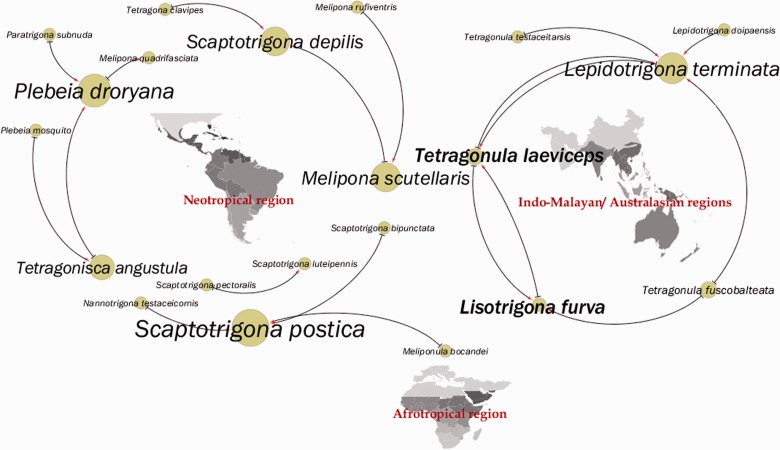
Network interactions among males of different stingless bee species sharing mixed-species reproductive aggregations noted in the literature. Notice that although males may aggregate with closely related species, they may also visit more distantly related stingless bee species. Origin of lines (⊤) indicates those stingless bee species (alien) whose males visited nonspecific (host) aggregations (red arrows). The larger the circle and the letter most frequently species was observed receiving or visiting other stingless bee species.

Overall, we found 14 Neotropical, one Afrotropical, and six Indo-Malayan/Australasian stingless bee species visiting in any time aggregations of another species (Fig. 2, Supp Table 1 [online only]). Males of the Neotropical, *Scaptotrigona* spp. and Indo-Malayan/Australasia, *Tetragonula* spp. and *Lepidotrigona* spp. showed the most records in the literature of individuals visiting nonspecific aggregations or receiving alien stingless bee species in their reproductive aggregations. Only *Scaptotrigona*, *Melipona,* and *Lepidotrigona* have been reported, to date, of receiving the visit of closely related genera males (references in Supp Table 1 [online only]). The oddest observation was when an Afrotropical male stingless bee, *Melipona bocandei* (Spinola), was recorded over five consecutive days in a male aggregation of the Neotropical bee *Scaptotrigona postica* (Latreille) (Fig. 2, Supp Table 1 [online only]).

During this study, we also observed two other uncommon behaviors. In September 2009, we made a video recording of a single male *M. scutellaris* visiting and peacefully interacting with a reproductive aggregation of *Scaptotrigona* aff. *depilis* males at the apiary of the Faculdade de Filosofia, Ciências e Letras de Ribeirão Preto, a part of the University of São Paulo, in Ribeirão Preto (Supp Video 1 [online only]). Again in July 2011, in the Universidade de São Paulo’s apiary, São Paulo, we recorded more than 30 *T. clavipes* males landing throughout the day on three consecutive days at one colony *S.* aff. *depilis* (Supp Fig. 3 [online only]). However, the *S.* aff. *depilis* males were absent therein but they could be seen aggregating in front of a colony of their species 20 m away.

#### Analysis of CHCs Profiles

Analyses of male bee CHCs showed a total of 45 hydrocarbons, which varied in combinations among species. They were classified as linear alkanes, alkenes, alkadienes and methyl-branched alkanes and ranged between 21 and 33 carbon atoms (Table 1). Some hydrocarbons were present in only one or two bee groups. The most abundant compounds that were common to all groups were pentacosane, heptacosane, and nonacosane. The species with the least number of compounds was *T. angustula* (17 hydrocarbons). Males of *Plebeia* sp. and *S.* aff. *depilis* had 18 CHC compounds, while males of *T. clavipes* had 19 compounds. *N. testaceicornis*, *M. subnitida,* and *T. spinipes* showed the highest number of CHCs: 28, 31, and 33, respectively.

**Table 1.** Relative concentrations (mean, SD) of CHCs in males of *Plebeia* sp., *T. clavipes*, *N. testaceicornis*, *M. subnitida*, *T. spinipes, T. angustula,* and *S.* aff. *depilis*

Hydrocarbons	RT (min)	*Plebeia pugnax*	*T. clavipes*	*N. testaceicornis*	*M. subnitida*	*T. spinipes*	*T. angustula*	*S. aff. depilis*
Heneicosane (*n*-C_21_)	18.054	0.11 ± 0.09	0.26 ± 0.35	—	0.09 ± 0.04	—	—	—
Docosane (*n*-C_22_)	19.944	—	—	—	—	—	0.09 ± 0.08	—
Tricosene^*^	22.765	—	—	—	0.22 ± 0.50	0.37 ± 0.33	—	—
Tricosane (*n*-C_23_)	23.546	3.93 ± 1.33	1.35 ± 0.66	3.42 ± 0.54	1.77 ± 0.92	1.87 ± 0.92	0.28 ± 0.20	0.82 ± 0.29
Tetracosene^*^	25.444	—	0.21 ± 0.56		—	0.22 ± 0.25	—	—
Tetracosane (*n*-C_24_)	26.199	—	0.80 ± 0.28	0.11 ± 0.09	0.45 ± 0.11	0.50 ± 0.15	0.32 ± 0.12	0.46 ± 0.10
7-Methyl C_24_	26.897	0.74 ± 0.28	—	0.50 ± 0.13	0.38 ± 0.16	—	—	—
Pentacosene^*^^a^	28.073	—	—	0.14 ± 0.17	1.14 ± 0.83	1.05 ± 0.30	—	0.53 ± 0.41
Pentacosene^*^^b^	28.291	—	—	0.09 ± 0.10	0.06 ± 0.05	0.32 ± 0.11	—	—
Pentacosene^*^^c^	28.419	0.54 ± 0.99	—		0.07 ± 0.08	—	—	—
Pentacosane (*n*-C_25_)	28.836	19.57 ± 6.32	50.07 ± 4.23	13.03 ± 1.83	25.94 ± 4.07	30.40 ± 6.16	46.78 ± 7.32	21.88 ± 3.04
13-; 11- Methyl C_25_	30.137	—	0.15 ± 0.13	1.37 ± 0.41	—	—	—	—
5-Methyl C_25_	30.500	—	—	0.15 ± 0.18	—	—	—	—
3- Methyl C_25_	31.299	—		0.53 ± 0.94	—	—	—	—
Hexacosene^*^	30.610	—	0.13 ± 0.07	—	0.11 ± 0.03	—	—	—
Hexacosane (*n*-C_26_)	31.300	—	0.74 ± 0.12	0.45 ± 0.12	0.78 ± 0.09	1.33 ± 0.13	1.45 ± 0.14	0.90 ± 0.17
Heptacosene^*^^a^	33.090	1.29 ± 0.69	—	3.83 ± 7.78	2.71 ± 0.70	2.60 ± 0.65	0.55 ± 0.63	24.00 ± 3.48
Heptacosene^*^^b^	33.282	—	0.92 ± 0.23	0.22 ± 0.18	0.18 ± 0.09	0.30 ± 0.10	—	0.96 ± 0.16
Heptacosene^*^^c^	33.745	—	—	—	0.11 ± 0.03	—	—	—
Heptacosane (*n*-C_27_)	33.773	10.21 ± 2.46	18.81 ± 1.38	19.89 ± 2.38	18.22 ± 1.95	46.18 ± 3.50	31.66 ± 2.92	14.37 ± 2.53
13-Methyl C_27_	34.850	—	—	2.49 ± 0.74	—	0.14 ± 0.07		0.73 ± 0.35
9-Methyl C_27_	35.020	—	—	0.12 ± 0.12	—	—	0.09 ± 0.21	—
7-Methyl C_27_	35.448	—	—	0.18 ± 0.14	—	—		—
Octacosene	35.474	—	0.50 ± 0.65	1.56 ± 0.21	0.88 ± 0.12	—	—	—
Octacosane (*n*-C_28_)	36.089	—	0.70 ± 0.10	0.18 ± 0.14	0.45 ± 0.09	0.53 ± 0.11	0.68 ± 0.15	0.68 ± 0.13
Nonacosadiene^*^^a^	37.134	—	—	0.13 ± 0.12	1.35 ± 0.60	0.20 ± 0.08	—	—
Nonacosadiene^*^^b^	37.300	—	—	—	0.23 ± 0.10	—	—	—
Nonacosene^*^^a^	37.605	18.78 ± 7.81	—	—	0.29 ± 0.11	—	—	—
Nonacosene^*^^b^	37.859	6.60 ± 3.97	0.10 ± 0.11	37.34 ± 5.81	25.87 ± 3.89	0.63 ± 0.16	0.75 ± 1.59	15.29 ± 6.12
Nonacosene^*^^c^	37.995	—	0.13 ± 0.06	1.29 ± 0.25	0.57 ± 0.53	0.15 ± 0.09	—	—
Nonacosane (*n*-C_29_)	38.378	5.83 ± 1.81	15.88 ± 2.85	3.31 ± 0.48	6.93 ± 1.68	8.44 ± 2.40	9.15 ± 1.98	8.97 ± 3.39
13-; 11-Methyl C_29_	39.030	0.29 ± 0.51	3.41 ± 2.89	0.94 ± 0.30	0.19 ± 0.08	0.10 ± 0.07	—	0.69 ± 0.97
Triacontene^*^	40.085	—	—	0.10 ± 0.11	0.41 ± 0.08	0.46 ± 0.44	—	—
Triacontane (*n*-C_30_)	40.585	—	0.39 ± 0.12	0.15 ± 0.25	0.07 ± 0.04	0.95 ± 0.68	0.51 ± 0.17	0.27 ± 0.13
?-Methyl C_30_	40.620	—	—	—	—	—	0.09 ± 0.14	—
Hentriacontadiene^*^^a^	41.506	0.91 ± 0.49	—	0.27 ± 0.26	1.22 ± 0.43	0.53 ± 0.26	—	—
Hentriacontadiene^*^^b^	41.613	0.47 ± 0.30	—	3.28 ± 1.08	0.54 ± 0.22	0.26 ± 0.21	—	—
Hentriacontene^*^^a^	41.956	16.15 ± 3.41	—	0.69 ± 0.40	0.29 ± 0.11	0.19 ± 0.22	—	5.24 ± 1.81
Hentriacontene^*^^b^	42.141	5.98 ± 1.27	—	2.26 ± 0.50	—	0.98 ± 0.54	—	—
Hentriacontene^*^^c^	42.227	6.70 ± 2.55	0.30 ± 0.72	0.85 ± 0.60	7.67 ± 1.59	0.10 ± 0.07	—	—
Hentriacontane (*n*-C_31_)	42.716	1.03 ± 0.43	5.16 ± 1.87	0.47 ± 0.07	0.85 ± 0.53	0.78 ± 0.70	6.44 ± 2.54	2.87 ± 5.23
?-Methyl C_31_	42.022	—	—	0.26 ± 0.21	—	0.30 ± 0.16	0.62 ± 0.65	—
Dotriacontane (*n*-C_32_)	43.449	—	—	—	—	—	0.26 ± 0.17	1.05 ± 2.87
Tritriacontene^*^	46.589	0.90 ± 0.52	—	0.39 ± 0.30	—	0.09 ± 0.06	—	—
Tritriacontane (*n*-C_33_)	44.640	—	—	—	—	—	0.12 ± 0.12	0.27 ± 0.28

^*^Double bonds not identified.

RT, retention time (min).

**Fig. 3.**
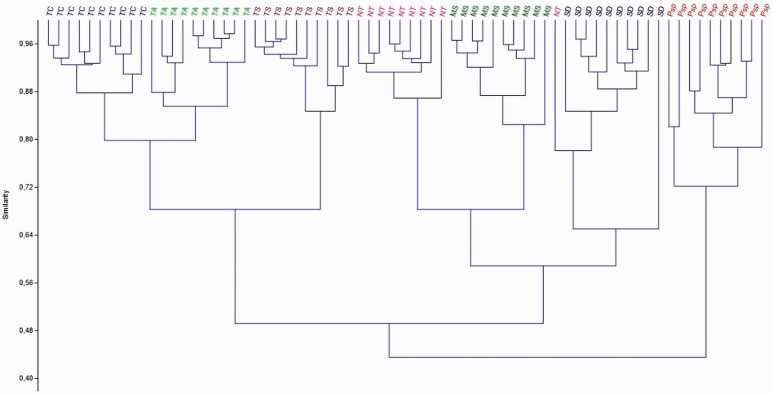
Cluster analysis using the Bray-Curtis similarity measure. TC, *Tetragona clavipes*; TA, *Tetragonisca angustula*; TS, *Trigona spinipes*; NT, *Nanotrigona testaceicornis*; MS, *Melipona subnitida*; SD, *Scaptotrigona* aff. *depilis*; PP, *Plebeia* sp.

The Bray-Curtis similarity cluster analysis shows that all individuals are correctly allocated to their predicted group, except for a single male of *N. testaceicornis* (Fig. 3). According to the cluster analysis, males of *T. clavipes* were more similar to those of *T. angustula*, and males of *N. testaceicornis* were more similar to those of *M. subnitida*. *T. spinipes* males were similar to *T. clavipes* and *T. angustula* males. Finally, *S.* aff. *depilis* males were similar to *N. testaceicornis,* and *M. subnitida*. *Plebeia* sp. was the group that showed the highest distance among the other species (Fig. 3).

**Fig. 4.**
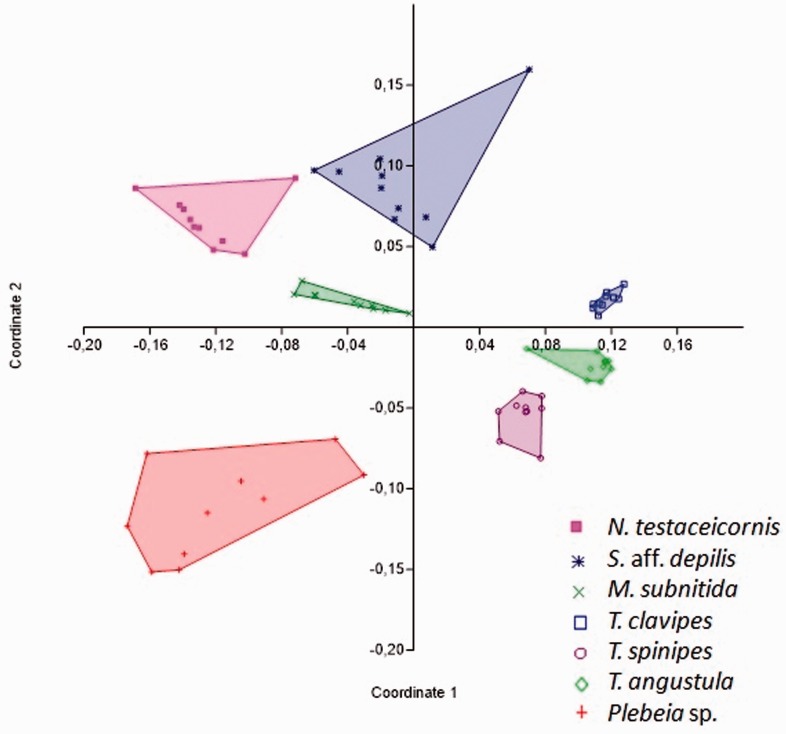
NMDS results showing the differences in the chemical profiles of CHCs of male stingless bees.

NMDS demonstrated that males showed species-specific differences in their chemical profiles (Fig. 4). The NMDS plot showed a lower dissimilarity among males of *T. clavipes*, *T. angustula,* and *T. spinipes* and males of *N. testaceicornis*, *M. subnitida,* and *S.* aff. *depilis*. In contrast, males of *Plebeia* sp. were well separated from the other groups of males in the NMDS plots, thus supporting the Bray-Curtis similarity measure.

ANOSIM showed that the CHC profiles of males exhibited large dissimilarities, with global *R* = 0.9632, *P* < 0.0001 (Table 2). The values for the Bray-Curtis dissimilarity indices in the CHC profiles of pairs of males were >0.90, except for *T. clavipes* versus *T. angustula* (*R* = 0.8769). *R* and *P* values in ANOSIM and the compounds that predominantly contribute to the Bray-Curtis dissimilarities are shown in Table 2.

**Table 2.** Results of ANOSIM and SIMPER analysis of the cuticular compounds of Meliponini males

	*R*-values	*P*-values	Compounds that predominantly contribute to Bray-Curtis dissimilarities
*P. pugnax* vs *T. clavipes*	1.0	< 0.0021	*n*-C_25_, Nonacosene^*a^, Hentriacontene^*a^
*P. pugnax* vs *N. testaceicornis*	1.0	< 0.0021	Nonacosene^*b^, Nonacosene^*a^, Hentriacontene^*a^
*P. pugnax* vs *M. subnitida*	1.0	< 0.0021	Nonacosene^*b^, Nonacosene^*a^, Hentriacontene^*a^
*P. pugnax* vs *T. spinipes*	1.0	<0.0021	Nonacosene^*a^, Hentriacontene^*a^, *n*-C_25_
*P. pugnax* vs *T. angustula*	1.0	<0.0021	*n*-C_25_, *n*-C_27_, Nonacosene^*a^, Hentriacontene^*c^
*P. pugnax* vs *S. depilis*	0.9969	<0.0021	Heptacosene^*a^, Nonacosene^*a^, Hentriacontene^*a^
*T. clavipes* vs *N. testaceicornis*	1.0	<0.0021	Nonacosene^*b^, *n*-C_25_, *n*-C_29_
*T. clavipes* vs *M. subnitida*	1.0	<0.0021	Nonacosene^*b^, *n*-C_25_, *n*-C_29_
*T. clavipes* vs *T. spinipes*	1.0	<0.0021	*n*-C_27_, *n*-C_25_
*T. clavipes* vs *T. angustula*	0.8769	<0.0021	*n*-C_27_, *n*-C_25_, *n*-C_29_
*T. clavipes* vs *S. depilis*	0.9973	<0.0021	*n*-C_25_, Heptacosene^*a^, Nonacosene^*b^
*N. testaceicornis* vs *S. depilis*	0.9609	<0.0021	Nonacosene^*b^, Heptacosene^*a^
*N. testaceicornis* vs *M. subnitida*	0.9078	<0.0021	*n*-C_25_, Nonacosene^*b^, Hentriacontene^*c^
*N. testaceicornis* vs *T. spinipes*	1.0	<0.0021	Nonacosene^*b^, *n*-C_27_, *n*-C_25_
*N. testaceicornis* vs *T. angustula*	1.0	<0.0021	Nonacosene^*b^, *n*-C_25_
*M. subnitida* vs *T. spinipes*	1.0	<0.0021	*n*-C_27_, Heptacosene^*b^
*M. subnitida* vs *T. angustula*	1.0	<0.0021	Nonacosene^*b^, *n*-C_25_, *n*-C_27_
*M. subnitida* vs *S. depilis*	0.902	<0.0021	Heptacosene^*a^, Nonacosene^*b^, Hentriacontene^*c^
*T. spinipes* vs *T. angustula*	0.9633	<0.0021	*n*-C_25_, *n*-C_27_, *n*-C_31_
*T. spinipes* vs *S. depilis*	0.9998	<0.0042	*n*-C_27_, Heptacosene^*a^, Heptacosene^*b^
*T. angustula* vs *S. depilis*	0.9989	<0.0021	*n*-C_25_, Heptacosene^*a^, *n*-C_27_, Nonacosene^*b^

*R* values between 0 and 1 indicate the level of similarity or dissimilarity (*R* = 0 indicates no difference between the species, *R* = 1 indicates a larger similarity within a group than between groups), *P* = Bonferroni adjusted *P*-values. * Different positions of double bonds (not identified).

## Discussion

Our literature review showed that, with few exceptions, alien male stingless bees visiting host species aggregations do not necessarily associate with closely related species (Schwarz 1948; Nogueira-Neto 1954; Kerr et al. 1962; Cortopassi-Laurino 1979, 2009; Bänziger and Khamyotchai 2014; Santos et al. 2014). It is known that phylogenetically more related stingless bee species tend to share similar CHCs profiles among themselves (Leonhardt et al. 2013). However, our analysis showed that despite it some stingless male bees may randomly aggregate together to distantly related species.

Our chemical analyses (NMDS and cluster analysis) provided evidence for a clear distinction in CHC profiles among the different stingless bee species analyzed. Thus, there is not any chemical overlap that could confuse males to aggregate with individuals of different stingless bee genera/species as suggested by Kerr et al. (1962). Nevertheless, there were some particular compounds (e.g., tricosane, pentacosane, heptacosane, and hentriacontane) which were common to the males of all the stingless bee species analyzed. However, such CHCs are rather common to almost all social insects and their functions seem to be more related to protection against desiccation, damage, and predators than having any function as a likely aggregation pheromone (Blomquist et al. 1998, Ayasse et al. 2001, Provost et al. 2008, Leonhardt et al. 2013).

To date, very little is known about the ability of male stingless bees to emit recruitment (aggregation) or sex pheromones and, thus, to attract partners for aggregations, albeit it has been suggested (Sommeijer et al. 2004, López and Kraus 2009). But, although none chemical experiment for attraction has been done here, our analysis of CHC profiles of different stingless bee species indicated that this is, as a whole, unlikely to explain the uncommon sexual behavior assumed by alien males. Future analysis testing behavioral responses between alien and host male odors may help us to better explain this uncommon behavior. We further suggest here another study possibility.

For example, it is known that *Trigonidium obtusum* and *Mormolyca ringens* orchids (Neotropical plant species) are chemically capable of mimicking some compounds (e.g., 2-alcohols and 9-alkene/alkane series) found in the sex queen pheromones of the stingless bees *N. testaceicornis*, *P. droryana,* and *Scaptotrigona* sp. attracting, then, these males for pseudocopulation (Singer 2002, Singer et al. 2004, Flach et al. 2006). Males of these stingless bee species, mentioned here, were already observed previously in mixed male aggregations (Kerr et al. 1962, Santos et al., 2014, Imperatriz-Fonseca apud Cortopassi-Laurino, 1979). Thus, sex queen pheromone of multiple stingless bee species could also be investigated by looking to see whether such pheromones could have any role in attracting different stingless bee males like have been observed for drones of different *Apis* species (Butler et al. 1967, Sannasi and Rajulu 1971, reviewed by Koeniger and Koeniger 2000).

Although cues causing attraction of alien males for reproductive aggregations of other stingless bee species are still unclear, this unexpected behavior may has deep implications for individual fitness of such males, as well as for its origin colonies. First, reproductive aggregations in stingless bees do not regularly occur throughout the year (Kerr et al. 1962, Cortopassi-Laurino 2007, Bänziger and Khamyotchai 2014, Santos et al. 2014). Therefore, males have few alternatives to choose correct aggregations to join during its short lifetime outside nests (2–3 wk). Second, although reproductive aggregations have thousands of individuals (Kerr et al. 1962, Santos et al. 2013, Bänziger and Khamyotchai 2014), the colonies usually send few males, oftentimes just one, for every aggregation (Paxton 2000, Cameron et al. 2004). It means that if such males adopt any mistaken behavior during this lifetime, it may not only compromise its own reproductive success estimated to be in the order of 0.01–0.002 (Velthuis et al. 2005) but also it to reduce still more chances of colonies (ultimately its mother queens) pass genes forward.

Stingless bee males are under strong sexual selection to find queens and copulate with them (Roubik 1990). The reproductive aggregations are extremely male-biased (Kerr et al. 1962, Cortopassi-Laurino 2007, Bänziger and Khamyotchai 2014, Santos et al. 2014) and only one can mate successfully (Kerr et al. 1962, Strassmann 2001, Jaffé et al. 2014). Therefore it is likely that morphological, physiological, and behavioral features should maximize the competitive ability and the male’s capacity to find and join conspecific aggregations. Nevertheless, alien stingless male bees seem to have lost their selective ability by adopting an apparently nonadaptive behavior. Furthermore, studies are needed to clarify this issue.

## Supplementary Data

Supplementary data are available at *Journal of Insect Science* online.

## References

[iev107-B1] Ayasse M. Paxton R. J. Tengö J. 2001 Mating behavior and chemical communication in the order Hymenoptera. Annu. Rev. Entomol. 46: 31–78. 11112163 10.1146/annurev.ento.46.1.31

[iev107-B2] Bänziger H. Khamyotchai K. 2014 An unusually large and persistent male swarm of the stingless bee *Tetragonula laeviceps* in Thailand (Hymenoptera: Apidae: Meliponini). J. Melittology 32: 1–5.

[iev107-B3] Blomquist G. J. Tillman J. A. Mpuru S. Seybold S. J. 1998 The cuticle and cuticular hydrocarbons of insects: structure, function, and biochemistry, pp. 35–54. *In* Vander Meer R. K. Breed M. D. Winston M. L. Espelie K. E. (eds.), Pheromone communication in social insects. Westview, Boulder.

[iev107-B4] Butler C. G. Calam D. H. Callow R. K. 1967 Attraction of *Apis mellifera* drones by the odours of the queens of two other species of honeybees. Nature 213: 423–424. 6067599 10.1038/213423a0

[iev107-B5] Cameron E. C. Franck P. Oldroyd B. P. 2004 Genetic structure of nest aggregations and drone congregations of the southeast Asian stingless bee *Trigona collina*. Mol. Ecol. 13: 2357–2364. 15245407 10.1111/j.1365-294X.2004.02194.x

[iev107-B6] Cortopassi-Laurino M 1979 Observações sobre atividades de machos de *Plebeia droryana* Friese (Apidae, Meliponinae). Rev. Bras. Entomol. 23: 177–191.

[iev107-B7] Cortopassi-Laurino M 2007 Drone congregations in Meliponini: what do they tell us? Biosci. J. 23: 153–160.

[iev107-B8] Cortopassi-Laurino M. 2009 O refúgio das abelhas “Paulo Nogueira-Neto.” Mensagem Doce. 3–5.

[iev107-B9] Dani F. R 2006 Cuticular lipids as semiochemicals in paper wasps and other social insects. Ann. Zool. Fennici. 43: 500–514.

[iev107-B10] Darwin C. R. 1859 On the origin of species. Or the preservation of favoured races in the struggle for life John Murray, London.

[iev107-B11] Emlen S. T. Oring L. W. 1977 Ecology, sexual selection, and the evolution of mating systems. Science 197: 215–223. 327542 10.1126/science.327542

[iev107-B12] Ferreira-Caliman M. J. Falcón T. Mateus S. Zucchi R. Nascimento F. S. 2013 Chemical identity of recently emerged workers, males, and queens in the stingless bee *Melipona marginata*. Apidologie 44: 657–665.

[iev107-B13] Ferreira-Caliman M. J. Nascimento F. S. Turatti I. C. Mateus S. Lopes N. P. Zucchi R. 2010 The cuticular hydrocarbons profiles in the stingless bee *Melipona marginata* reflect task-related differences. J. Insect Physiol. 56: 800–4. 20170657 10.1016/j.jinsphys.2010.02.004

[iev107-B14] Flach A. Marsaioli A. J. Singer R. B. Amaral M.C.E. Menezes C. Kerr W. E. Batista-Pereira L. G. Corrêa A. G. 2006 Pollination by sexual mimicry in *Mormolyca ringens*: a floral chemistry that remarkably matches the pheromones of virgin queens of *Scaptotrigona* sp. J. Chem. Ecol. 32: 59–70. 16525870 10.1007/s10886-006-9351-1

[iev107-B15] Hadley N. F 1994 Water relations of terrestrial arthropods. Academic Press Inc., London.

[iev107-B16] Hammer Ø. Harper D.A.T. Ryan P. D. 2001 PAST: Paleontological statistics software package for eduaction and data analysis. Version 2.15. Paleontol. Eletronica 4: 9.

[iev107-B17] Jaffé R. Pioker-Hara F. C. Santos C. F. Santiago L. R. Alves D. A. Kleinert A.M.P. Francoy T. M. Arias M. C. Imperatriz-Fonseca V. L. 2014 Monogamy in large bee societies: a stingless paradox. Naturwissenschaften 101: 261–264. 24463620 10.1007/s00114-014-1149-3

[iev107-B18] Kerr W. E. Zucchi R. Nakadaira J. T. Butolo J. E. 1962 Reproduction in the social bees (Hymenoptera: Apidae). J. N Y Entomol. Soc. 70: 265–276.

[iev107-B19] Koeniger N. Koeniger G. 2000 Reproductive isolation among species of the genus *Apis*. Apidologie 31: 313–339.

[iev107-B20] Leonhardt S. D. Rasmussen C. Schmitt T. 2013 Genes versus environment: geography and phylogenetic relationships shape the chemical profiles of stingless bees on a global scale. Proc. R. Soc. B Biol. Sci. 280 http://dx.doi.org/10.1098/rspb.2013.0680. 10.1098/rspb.2013.0680 PMC3673053 23658202

[iev107-B21] Leprince D. J. Francoeur A. 1986 Hilltop swarming by ants (Hymenoptera: Formicidae) in southwestern Quebec and interspecific competition at the swarm marker. Ann. Entomol. Soc. Am. 79: 865–869.

[iev107-B22] López J.C.G. Kraus F. B. 2009 Cherchez la femme? Site choice of drone congregations in the stingless bee *Scaptotrigona mexicana*. Anim. Behav. 77: 1247–1252.

[iev107-B23] Michener C. D 1946 Notes on the habits of some Panamanian stingless bees (Hymenoptera, Apidae). J. N Y Entomol. Soc. 54: 179–197.

[iev107-B24] Nadel H 1987 Male swarms discovered in *Chalcidoidea* (Hymenoptera: Encyrtidae, Pteromalidae). Pan-Pac. Entomol. 63: 242–246.

[iev107-B25] Nascimento D. L. Nascimento F. S. 2012 Acceptance threshold hypothesis is supported by chemical similarity of cuticular hydrocarbons in a stingless bee, *Melipona asilvai*. J. Chem. Ecol. 38: 1432–1440. 23053920 10.1007/s10886-012-0194-7

[iev107-B26] Nogueira-Neto P 1954 Notas bionômicas sobre meliponíneos: III–Sobre a enxameagem. Arq. do Mus. Nac. 19: 419–452.

[iev107-B27] Nunes T. M. Nascimento F. S. Turatti I. C. Lopes N. P. Zucchi R. 2008 Nestmate recognition in a stingless bee: does the similarity of chemical cues determine guard acceptance? Anim. Behav. 75: 1165–1171.

[iev107-B28] O’Neill K. M 1994 The male mating strategy of the ant *Formica subpolita* Mayr (Hymenoptera: Formicidae): swarming, mating, and predation risk. Psyche (Stuttg). 101: 93–108.

[iev107-B29] Paxton R. J 2000 Genetic structure of colonies and a male aggregation in the stingless bee *Scaptotrigona postica*, as revealed by microsatellite analysis. Insectes Soc. 47: 63–69.

[iev107-B30] Provost E. Blight O. Tirard A. Renucci M. 2008 Hydrocarbons and insects’ social physiology, pp. 19–72. *In* Maes R. P. (ed.), Insect physiology: new research. Nova Science Publishers, Inc., New York.

[iev107-B31] R Core Team. 2014 R: A language and environment for statistical computing. The R Foundation for Statistical Computing, Vienna, Austria [Computer software]. (http://www.R-project.org/) (accessed 18 January 2015).

[iev107-B32] Roubik D. W 1990 Mate location and mate competition in males of stingless bees (Hymenoptera: Apidae: Meliponinae). Entomol. Gen. 15: 115–120.

[iev107-B33] Saito R. Smoot M. E. Ono K. Ruscheinski J. Wang P.-L. Lotia S. Pico A. R. Bader G. D. Ideker T. 2012 A travel guide to Cytoscape plugins. Nat. Methods 9: 1069–1076. 23132118 10.1038/nmeth.2212 PMC3649846

[iev107-B34] Sannasi A. Rajulu G. S. 1971 9-oxodec-trans-2-enoic acid in the Indian honeybees. Life Sci. 10: 195–201. 10.1016/0024-3205(71)90018-x 5575792

[iev107-B35] Santos C. F. Menezes C. Imperatriz-Fonseca V. L. Arias M. C. 2013 A scientific note on diploid males in a reproductive event of a eusocial bee. Apidologie 44: 519–521.

[iev107-B36] Santos C. F. Menezes C. Vollet-Neto A. Imperatriz-Fonseca V. L. 2014 Congregation sites and sleeping roost of male stingless bees (Hymenoptera: Apidae: Meliponini). Sociobiology 61: 115–118.

[iev107-B37] Schwarz H. F. 1948 Stingless bees (Meliponidae) of the Western Hemisphere: *Lestrimelitta* and the following subgenera of *Trigona*: *Trigona*, *Paratrigona*, *Schwarziana*, *Parapartamona*, *Cephalotrigona*, *Oxytrigona*, *Scaura*, and Mourella. Bulletin of the American Museum of Natural History, New York.

[iev107-B38] Shannon P. Markiel A. Ozier O. Baliga N. S. Wang J. T. Ramage D. Amin N. Schwikowski B. Ideker T. 2003 Cytoscape: a software environment for integrated models of biomolecular interaction networks. Genome Res. 13: 2498–2504. 14597658 10.1101/gr.1239303 PMC403769

[iev107-B39] Singer R. B 2002 The pollination mechanism in *Trigonidium obtusum* Lindl (Orchidaceae: Maxillariinae): sexual mimicry and trap-flowers. Ann. Bot. 89: 157–163. 12099346 10.1093/aob/mcf021 PMC4233788

[iev107-B40] Singer R. B. Flach A. Koehler S. Marsaioli A. J. Amaral M.C.E. 2004 Sexual mimicry in *Mormolyca ringens* (Lindl.) Schltr. (Orchidaceae: Maxillariinae). Ann. Bot. 93: 755–762. 15051623 10.1093/aob/mch091 PMC4242296

[iev107-B41] Sivinski J. M. Petersson E. 1997 Mate choice and species isolation in swarming insects, pp. 294–309. *In* Choe J. C. Crespi B. J. (eds.), Evolution of mating systems in insects and *Arachnids*. Cambridge University Press, UK, USA, Australia.

[iev107-B42] Sommeijer M. J. Bruijn L.L.M. Meeuwsen F. 2004 Behavior of males, gynes and workers at drone congregation sites of the stingless bee *Melipona favosa* (Apidae: Meliponini). Entomolosgische Berichtein 64: 10–15.

[iev107-B43] Starr C. K. Vélez D. 2009 A Dense daytime aggregation of solitary bees (Hymenoptera: Apidae: Centridini) in the lesser antilles. J. Hymenopt. Res. 18: 175–177.

[iev107-B44] Strassmann J 2001 The rarity of multiple mating by females in the social Hymenoptera. Insectes Soc. 48: 1–13.

[iev107-B45] Sullivan R. T 1981 Insect swarming and mating. Florida Entomol. 64: 44–65.

[iev107-B46] Tannure-Nascimento I. C. Nascimento F. S. Zucchi R. 2008 The look of royalty: visual and odour signals of reproductive status in a paper wasp. Proc. R. Soc. B Biol. Sci. 275: 2555–2561. 10.1098/rspb.2008.0589 PMC2605800 18682372

[iev107-B47] Thornhill R. Alcock J. 1983 The evolution of insect mating systems. Harvard University Press, Cambridge, MA, 547 pp.

[iev107-B48] Velthuis H. H. W. Koedam D. Imperatriz-Fonseca V. L. 2005 The males of *Melipona* and other stingless bees, and their mothers. Apidologie 36: 169–185.

[iev107-B49] Wigglesworth V. B 1957 The physiology of insect cuticle. Annu. Rev. Entomol. 2: 37–54.

[iev107-B50] Wilkinson L. 2011 venneuler: Venn and Euler diagrams. (R package version 1.1-0) [Computer software]. (http://CRAN.R-project.org/package=venneuler) (accessed 18 January 2015).

